# Inhibition of the enzyme activity of cytochrome P450 1A1, 1A2 and 3A4 by fucoxanthin, a marine carotenoid

**DOI:** 10.3892/ol.2013.1457

**Published:** 2013-07-12

**Authors:** YOSHIKO SATOMI, HOYOKU NISHINO

**Affiliations:** 1 Faculty of Pharmaceutical Sciences, Suzuka University of Medical Science, Suzuka, Mie 513-8670, Japan; 2 Kyoto Prefectural University of Medicine, Kawaramachi-Hirokoji, Kamigyo-ku, Kyoto 602-8566, Japan; 3 Ritsumeikan University, Noji-Higashi, Kusatsu, Shiga 525-8577, Japan

**Keywords:** fucoxanthin, carotenoid, cytochrome P450, cancer prevention

## Abstract

Fucoxanthin is a carotenoid that is mainly identified in brown algae and is known to have anticarcinogenic and anti-tumor activities. Carotenoids have generally been shown to induce the expression and enzyme activity of the cytochrome P450s (CYPs). The present study evaluated the effect of fucoxanthin on the expression and enzymatic activity of the major xenobiotic metabolizing enzymes, CYP1A1, CYP1A2 and CYP3A4. Fucoxanthin markedly induced the expression of *cyp1a1* mRNA in HepG2 cells, but inhibited its enzyme activity in the cells and *in vitro*. Fucoxanthin also inhibited the enzyme activity of CYP1A2 and CYP3A4 in a dose-dependent manner *in vitro*. These results suggest that fucoxanthin may serve as a useful agent in cancer prevention with less adverse effects than β-carotene, including the activation of pro-carcinogens by CYPs.

## Introduction

Epidemiological and animal studies have shown that carotenoids possess cancer preventive and tumor inhibitory activities. However, clinical trials using β-carotene have resulted in the induction of lung cancer in male heavy smokers and asbestos workers, thus raising complex questions pertaining to the properties of carotenoids (1). Extensive research concerning the action of carotenoids is required in order to elucidate the effects of carotenoids on human health. Although the mechanism by which β-carotene increases the incidence of lung cancer remains to be elucidated, it has been speculated that the expression of the cytochrome P450 (CYP) enzymes, including CYP1A1/2 and CYP3A, that is induced by β-carotene may be responsible for the activation of pro-carcinogens and thus, the increase in the risk of lung cancer (2).

The CYP complex was originally noted for its heme-containing mono-oxygenase activity and has been implicated in the biosynthesis of steroid hormones and the oxidative metabolism of drugs (3,4). Of the CYP family of enzymes, CYP1A1, 1A2, 1B1, 2A6, 2A13, 2E1 and 3A4 are considered to be the main enzymes that are involved in carcinogen metabolism (4–6). CYP1A1 is expressed in numerous tissues, including the lungs, mammary glands and placenta, but is almost absent in the liver, while, in contrast, CYP1A2 is mainly identified in the liver. CYP1A1 and 1A2 are involved in the activation of pro-carcinogens through the aryl hydrocarbon receptor and the detoxification of carcinogens, and CYP1A2 is also involved in the metabolism of certain drugs, including caffeine and theophylline (6–8). CYP3A4 is a major enzyme that is involved in drug metabolism and also in the activation of certain pro-carcinogens, including aflatoxin B1 (6,9,10).

Carotenoids have generally been shown to induce the expression and enzyme activity of CYPs (11–14). Certain studies have shown that carotenoids inhibit the activity of CYPs (15–17) and are involved in the detoxification of pro-carcinogens, including aflatoxin B1 (18). Therefore, although it has been speculated that CYPs are involved in the action of carotenoids, particularly in association with carotenoid anticarcinogenic activity, the nature of this involvement is yet to be clarified. Fucoxanthin is a carotenoid that is mainly identified in brown algae and is known to have anticarcinogenic and tumor inhibitory activity (19–23). Since the effect of fucoxanthin on CYPs is unknown, the present study evaluated the effects of fucoxanthin on the expression and enzymatic activity of CYPs.

## Material and methods

### Chemicals

**Figure 1:**
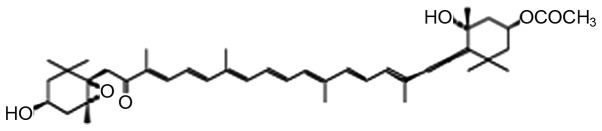
Structure of fucoxanthin.

Fucoxanthin (Fig. 1) was isolated as previously described (23) and was obtained from Dr Yoshito Tanaka (Faculty of Fisheries, Kagoshima University, Japan). The fucoxanthin was dissolved in dimethyl sulfoxide. Positive controls, 3-methylchoranthrene (3-MC), a known CYP1A1/2 inducer, omeprazole, a known CYP1A1 inhibitor, α-naphthoflavone, a known CYP1A2 inhibitor and ketoconazole, a known CYP3A4 inhibitor, were purchased from Sigma-Aldrich (Tokyo, Japan). 7-Ethoxyresorufin was also purchased from Sigma-Aldrich. All other chemicals were of biological grade.

### Cell culture

Human hepatocellular carcinoma HepG2 cells were cultured in Dulbecco's modified Eagle's medium (DMEM) supplemented with 10% heat-inactivated fetal bovine serum (FBS). The cells were maintained in an incubator at 37°C under a humidified atmosphere comprising 5% CO_2_. The cell viability was determined using the trypan blue dye exclusion test.

### Northern blot analysis

Total RNA was extracted from the cells. A total of 20 μg total RNA was electrophoresed and then transferred to a nylon membrane. Northern blots were hybridized with a ^32^P-labeled probe. PCR was performed to generate a cDNA probe for the *cyp1a1* gene (GenBank accession no. NM_00049). The data was normalized to the level of *36b4* expression.

### Measurement of CYP1A1/2 activity in HepG2 cells

The cells were plated at a density of 3×10^4^ cells/100 μl medium in 96-well plates. Subsequent to 24 h, the cells were treated with fucoxanthin or other chemicals, including phytoene and β-carotene for another 24 h. CYP activity was determined using the P450-Glo CYP1A1 assay (Promega KK, Tokyo, Japan), according to the manufacturer's instructions, and the 7-ethoxyresorufin-*O*-deethylase (EROD) assay. For the P450-Glo assay, the cells were washed twice with phosphate-buffered saline without Ca^2+^ and Mg^2+^ (PBS; -), and serum-free medium containing 50 μM luminogenic CYP1A1-specific substrate and luciferin-6′-chloroethyl ether was added. The cells were incubated at 37°C for 3 h, following which, 50 μl medium from each well was mixed with a luciferin detection reagent and the mixture was incubated at room temperature for 20 min. The luminescence was measured using a SpectraMax M5 microplate reader (Molecular Devices, Tokyo, Japan). The EROD assays were performed as previously described (24). Briefly, the cells were washed twice with PBS (−), and then 5 μM ethoxyresorufin and 1.5 mM salicylamide in a serum-free medium were added. Following incubation for 1 h at 37°C, the fluorescence was measured, with excitation at 560 nm and emission at 595 nm, using a SpectraMax M5 microplate reader. The CYP activity was expressed as luminescence/protein (P450-Glo assay) or fluorescence/protein (EROD assay) in each sample. The protein content of the samples was determined using a BCA Protein Assay kit (Pierce, Rockford, IL, USA).

### Measurement of CYP1A1/2 and CYP3A4 activity in vitro

CYP1A1 activity was determined using the P450-Glo CYP1A1 assay (Promega KK) and CYP1A2 and CYP3A4 activities were determined using Vivid CYP450 Screening kits (Life Technologies, Tokyo, Japan), according to the manufacturer's instructions. Microsomes containing the human CYP1A1 isozyme (recombinant) were purchased from Sigma-Aldrich. For the P450-Glo assays, a 50 μl reaction mixture containing 0.5 pmol CYP1A1, 400 mM KPO_4_ buffer and 120 mM luciferin-6′-chloroethyl ether, a luminogenic CYP1A1-specific substrate, was mixed with an equal volume of test compound and the mixture was incubated at room temperature for 10 min. Following this, an equal volume of NADPH regenerating system solution (2.6 mM NADP^+^, 6.6 mM glucose-6-phosphate, 6.6 mM MgCl_2_ and 0.8 U/ml glucose-6-phosphate dehydrogenase) was added. The reaction mixture was incubated at room temperature for 30 min. Luciferin detection reagent was then added and the mixture was incubated at room temperature for 20 min. The luminescence was then measured using a microplate reader. For the Vivid CYP450 Screening kit assay, the test compound was mixed with a master pre-mix comprising CYP450 BACULOSOMES^®^ reagent and regeneration system, which contained glucose-6-phosphate and glucose-6-phosphate dehydrogenase. The mixture was incubated at room temperature for 20 min. Following incubation, each CYP enzyme-specific substrate (Vivid EOMCC for CYP1A2 or Vivid DBOMF for CYP3A4) and NADP^+^ were added and the mixture was incubated at room temperature for 30 min. The reaction was stopped by the addition of 3 μM α-naphthoflavone (for CYP1A2) or 10 mM ketoconazole (for CYP3A4). CYP activity was evaluated by measuring the fluorescence of the fluorescent metabolite generated from each CYP enzyme-specific substrate. The fluorescence was measured using a microplate reader.

### Statistical analysis

The data were analyzed using Student's t-test. P<0.05 was considered to indicate a statistically significant difference.

## Results

### Inductive effect of fucoxanthin on the expression of the cyp1a1 gene in HepG2 cells

**Figure 2:**
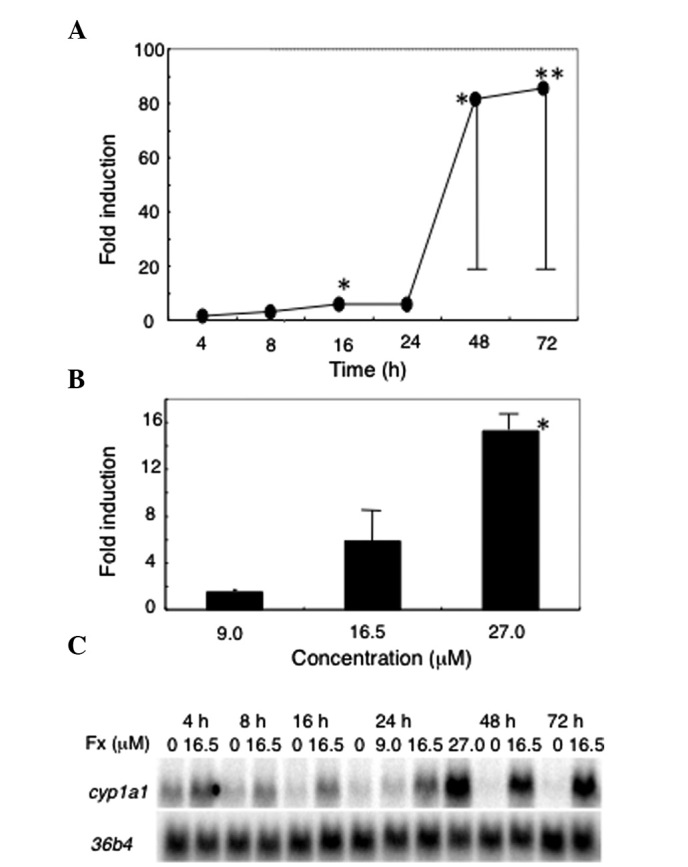
Effect of fucoxanthin on *cyp1a1* gene expression. (A) The cells were treated with 16.5 μM fucoxanthin for the indicated time. (B) The cells were treated with fucoxanthin (9.0, 16.5 and 27.0 μM) for 24 h. The expression of *cyp1a1* was normalized to that of *36b4*. (C) Representative data for *cyp1a1* expression as determined by northern blot analysis. Cyp, cytochrome P450; Fx, Fucoxanthin. Data are expressed as the mean ± SD (n=3). ^*^P<0.05 and ^**^P<0.01 vs. the control.

Fucoxanthin dramatically induced the expression of *cyp1a1* in a dose-dependent and time-dependent manner (Fig. 2). At 24 h post-treatment with 16.5 μM fucoxanthin, *cyp1a1* was induced 5.9-fold compared with the control. Fucoxanthin (16.5 μM) induced the expression of *cyp1a1* as early as 8 h after the treatment (not significant) and the inductive effect increased markedly at 48 h and continued up to 72 h (85.6-fold compared with the control). *cyp1a2* was not enhanced (data not shown) and *cyp3a4* was not expressed in the HepG2 cells.

### Inhibitory effect of fucoxanthin on CYP1A1/2 enzyme activity in HepG2 cells

**Figure 3:**
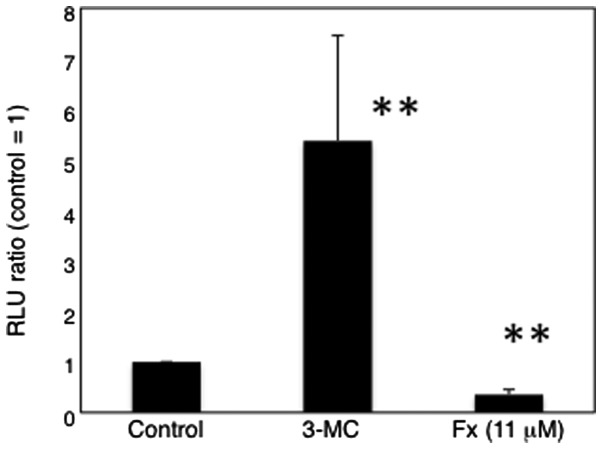
Effect of fucoxanthin on the activity of CYP1A1 in HepG2 cells. The cells were treated with fucoxanthin for 24 h and then a P450-Glo assay was performed. 3-MC (2.5 μM) was used as a positive control. 3-MC, 3-methylchoranthrene; Fx, fucoxanthin; RLU, relative luminescence unit. Data are expressed as the mean ± SD (n=2–5). ^**^P<0.01 vs. the control.

**Figure 4:**
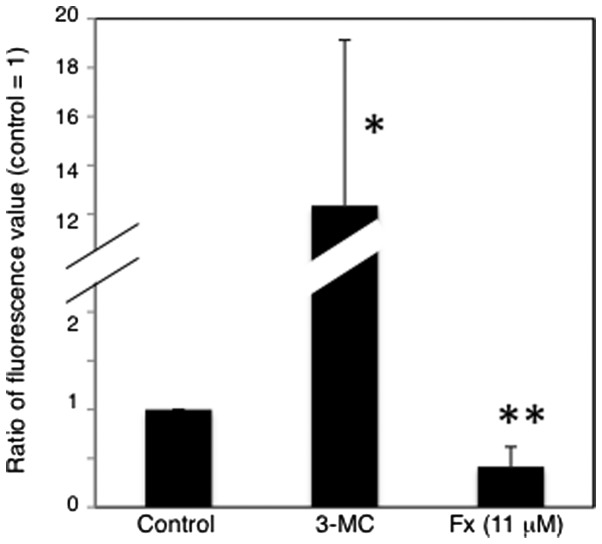
Effect of fucoxanthin on the EROD activity in HepG2 cells. The cells were treated with fucoxanthin for 24 h and the EROD activity was measured. 3-MC (2.5 μM) was used as a positive control. Fx, fucoxanthin; 3-MC, 3-methylchoranthrene; EROD, 7-ethoxyresorufin-*O*-deethylase. Data are expressed as the mean ± SD (n=3–5). ^**^P<0.01 and ^*^P<0.05 vs. the control.

Fucoxanthin (11 μM) significantly decreased CYP1A1 enzyme activity in the HepG2 cells (Fig. 3). In contrast, 3-MC, a known *cyp1a1* gene inducer, significantly induced CYP1A1 enzyme activity, while other carotenoids, including phytoene and β-carotene, demonstrated no inhibitory effect on CYP1A1 enzyme activity (data not shown). Furthermore, the EROD assay, which measures CYP1A1 and CYP1A2 activity, revealed the same results (Fig. 4). The data suggest that fucoxanthin is able to inhibit the activity of CYP1A1 and CYP1A2.

### Inhibitory effect of fucoxanthin on human recombinant CYP enzyme activity

**Figure 5:**
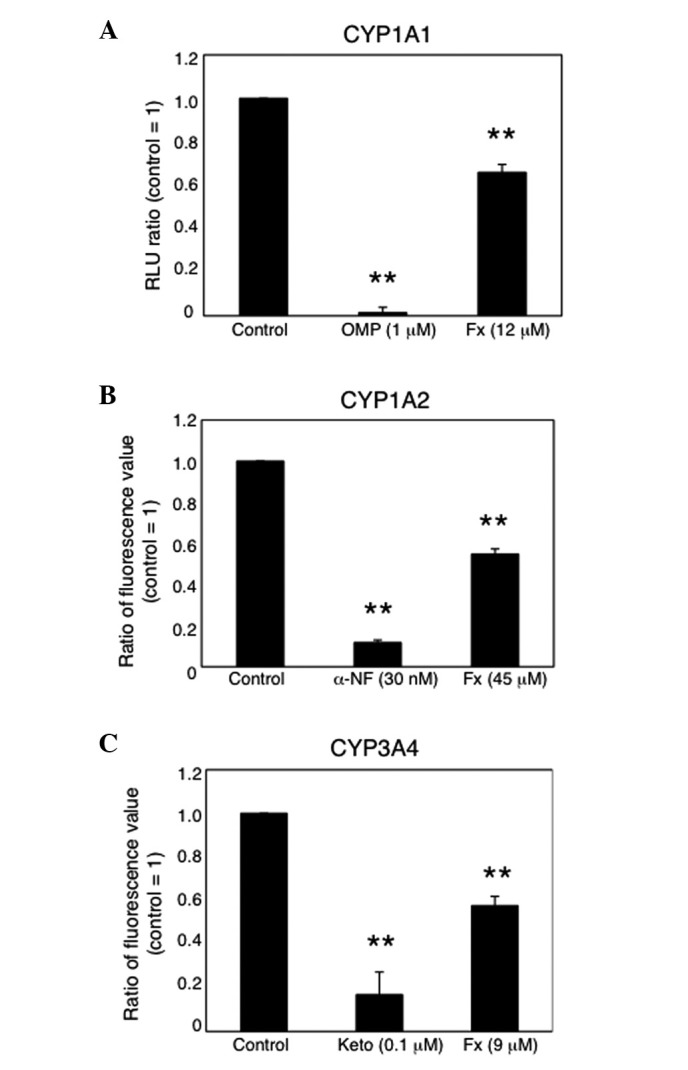
Effect of fucoxanthin on the activity of human recombinant CYP enzymes. (A) CYP1A1 activity was determined using the P450-Glo assay and microsomes containing recombinant human CYP1A1. (B) CYP1A2 and (C) CYP3A4 activity was determined using Vivid CYP450 Screening kits supplied with baculosomes containing each CYP. Omeprazole (1 μM), α-naphthoflavone (30 nM) and ketoconazole (0.1 μM) were used as positive controls for each CYP enzyme. CYP, cytochrome P450; Fx, fucoxanthin; OMP, omeprazole; α-NF, α-naphthoflavone; Keto, ketoconazole; RLU, relative luminescence unit. Data are expressed as the mean ± SD (n=3). ^**^P<0.01 vs. the control.

**Figure 6:**
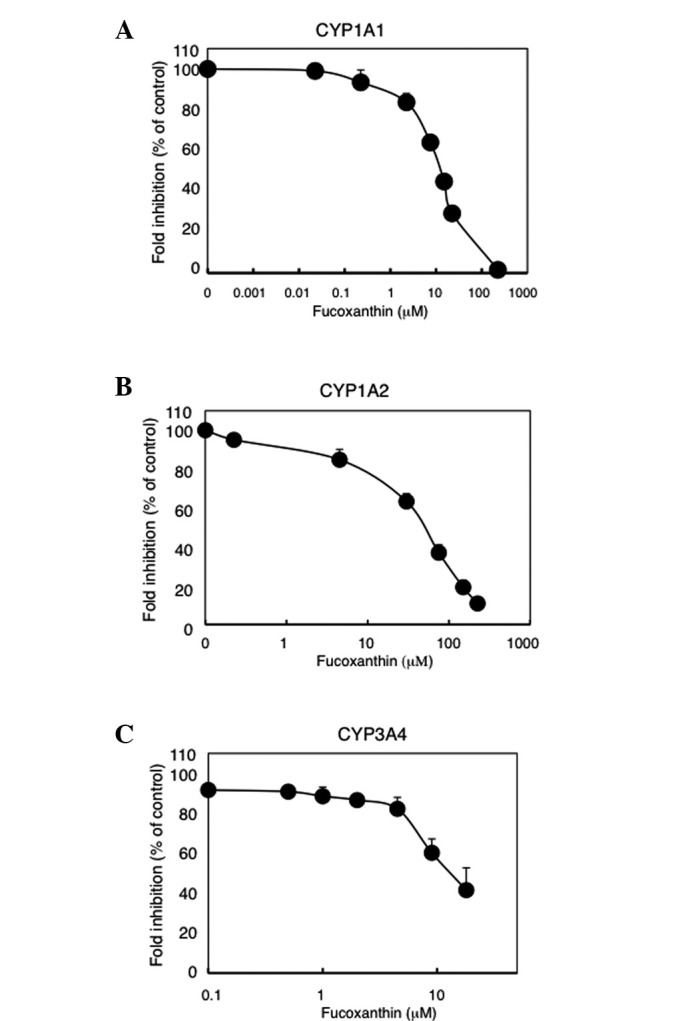
Inhibition of human recombinant CYP enzyme activity by fucoxanthin. (A) CYP1A1 activity was determined using the P450-Glo assay and microsomes containing recombinant human CYP1A1. (B) CYP1A2 and (C) CYP3A4 activity was determined using Vivid CYP450 Screening kits supplied with baculosomes containing each CYP. Data are expressed as the mean ± SD (n=2–4). CYP, cytochrome P450.

**Figure 7:**
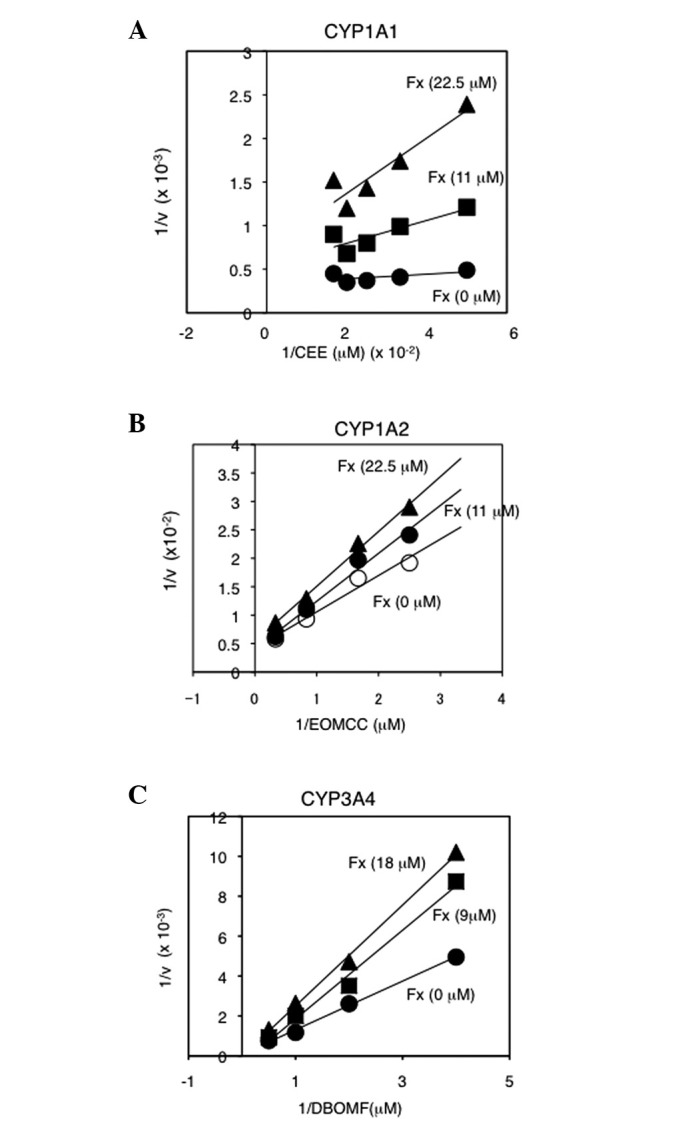
Lineweaver-Burk plots of CYP enzyme activity in the absence or presence of fucoxanthin. (A) CYP1A1 activity was determined using the P450-Glo assay and microsomes containing recombinant human CYP1A1. (B) CYP1A2 and (C) CYP3A4 activity was determined using Vivid CYP450 Screening kits supplied with baculosomes containing each CYP. The assays were conducted in the absence or presence of fucoxanthin and various concentrations of specific substrate for each CYP enzyme. EOMCC and DBOMF were employed as specific substrates for CYP1A2 and CYP3A4, respectively, supplied with Vivid CYP450 Screening kits. Typical data are shown. CYP, cytochrome P450; CEE, Luciferin-6′-chloroethyl ether.

The inhibition of CYP enzyme activity by fucoxanthin was measured using a specific substrate (Fig. 5). Fucoxanthin (12 μM) significantly inhibited CYP1A1 activity, although the inhibition was weaker than that of omeprazole, a known CYP1A1 inhibitor. Similarly, fucoxanthin (45 μM and 9 μM) significantly inhibited CYP1A2 and CYP3A4 activity, respectively, though the inhibition was weaker than that of α-naphthoflavone, a known CYP1A2 inhibitor, and of ketoconazole, a known CYP3A4 inhibitor. CYP1A1, CYP1A2 and CYP3A4 enzyme activity was inhibited by fucoxanthin in a dose-dependent manner, with IC50 values of 12.5, 49.0 and 11.0 μM, respectively (Fig. 6). The inhibitory effect of fucoxanthin on CYP1A2 was slightly weaker compared with CYP1A1 and CYP3A4. Lineweaver-Burk plots showed that fucoxanthin was not a competitive inhibitor for all the CYP enzymes that were examined (Fig. 7).

## Discussion

In an effort to ascertain the role of fucoxanthin in cancer prevention, the present study evaluated the effect of fucoxanthin on the enzyme activities of CYP1A1, 1A2 and 3A4, which are known to be involved in the activation of pro-carcinogens. Since carotenoids are known to induce phase I enzymes, including CYP1A1, they may enhance cancer initiation under certain conditions. The intake of β-carotene supplements has been reported to increase the incidence of lung cancer in male smokers and asbestos workers (1). One hypothesis put forward to account for this involved the induction of CYP enzymes by β-carotene (2,25).

In the present study, fucoxanthin significantly induced *cyp1a1* mRNA (Fig. 2), but inhibited CYP1A1 enzyme activity (Fig. 3), in the HepG2 cells. The EROD assay also demonstrated that fucoxanthin inhibited CYP1A1/2 activity, in HepG2 cells (Fig. 4). The data suggest that fucoxanthin may inhibit specific CYP enzyme activity, regardless of its inductive effect on expression.

The effect of fucoxanthin on the enzyme activity of recombinant human CYPs (CYP1A1, CYP1A2 and CYP3A4) was also analyzed *in vitro*. Fucoxanthin inhibited the activity of these CYPs, though the effect was weaker compared with known inhibitors, including omeprazole for CYP1A1, α-naphthoflavone for CYP1A2 and ketoconazole for CYP3A4 (Fig. 5). Few studies have examined the potential of carotenoids in the inhibition of CYP enzyme activity. Lycopene has been shown to inhibit the activity of CYP1A1 and CYP1B1 (17). Fucoxanthin has also been described as inhibiting CYP3A4 enzyme activity in HepG2 cells (15). However, the present study was unable to detect CYP3A4 activity or CYP3A4 mRNA expression in the HepG2 cells. Alterations in the nature of the cells utilized between laboratories may account for this discrepancy. In any case, fucoxanthin was shown to inhibit the activity of CYP1A1, CYP1A2 and CYP3A4.

The mechanism by which fucoxanthin inhibits the activity of the aforementioned CYPs remains to be delineated. Lineweaver-Burk plots revealed that fucoxanthin is not a competitive inhibitor of these CYPs (Fig. 7). Wang H *et al* showed that lycopene competitively inhibited CYP1A1 and CYP1B1 enzymes (17). Although diverse compounds may be acted upon by these CYPs, the main substrates of CYP1A1, CYP1A2 and CYP3A4 are polycyclic hydrocarbons, arylamines and steroids, respectively, and include a number of drugs. Thus, certain carotenoids may act as competitive inhibitors of CYPs. However, the present study did not observe that fucoxanthin was a competitive inhibitor of these CYPs. Fucoxanthin may attenuate CYP enzyme activity through an allosteric mechanism.

In conclusion, the present data suggested that fucoxanthin inhibits CYP1A1, CYP1A2 and CYP3A4 enzyme activity and that these inhibitory effects may contribute towards the cancer preventive action of fucoxanthin. The data also suggested that fucoxanthin may attenuate the action of certain drugs activated by CYP3A4 through the inhibition of CYP3A4. Further research is required with regard to the underlying mechanism of the inhibitory action of fucoxanthin on CYP enzyme activity.
